# Genetic Effects of *Soluble Starch Synthase IV-2* and It with *ADPglucose Pyrophorylase Large Unit* and *Pullulanase* on Rice Qualities

**DOI:** 10.1186/s12284-020-00409-0

**Published:** 2020-07-13

**Authors:** Liang Xu, Hui You, Ouling Zhang, Xunchao Xiang

**Affiliations:** Lab of Plant Molecular Genetics and Breeding, Southwest University of Science and Technology, 59 Qinglong Road, Mianyang, 621010 China

**Keywords:** *SSIV-2*, *AGPlar*, *PUL*, Epistatic effect, Rice quality, ECQs

## Abstract

**Background:**

Rice amylose content and amylopectin structure corporately determine rice eating and cooking qualities (ECQs). *Soluble starch synthase* (*SS*) *IV-2* is a member of the soluble starch synthesis gene family but with unknown effects on ECQs.

**Results:**

In this study, three populations derived from a cross of two parents who possess the same major genes of starch bio-synthesis were employed to investigate the influence of *SSIV-2* and its combined effects with *ADPglucose pyrophorylase large unit* (*AGPlar*) and *Pullulanase* (*PUL*) on ECQs. The results illustrated that the polymorphism of *SSIV-2* alleles significantly affected gel consistency (GC), gelatinization temperature (GT), percent of retrogradation (PR) and three crucial rapid viscosity analysis (RVA) profile parameters: peak viscosity (PKV), breakdown viscosity (BDV) and setback viscosity (SBV). And *SSIV-2* allele derived from CG173R had better quality traits with lower GT, SBV and PR. Moreover, its interaction with *AGPlar* was responsible for the variations of GC, apparent amylose content (AAC), GT, PR and all RVA parameters except for pasting temperature (PaT) and peak time (PeT), in terms of GC, PKV and CSV, *AGPlar* derived from CG173R had an epistatic effect on *SSIV-2*; additionally, interaction of *SSIV-2* and *PUL* mainly affected GC, AAC, PKV, CPV, CSV and SBV. I-C and C-1 (I, allele of *AGPlar* from Guangzhan 63S; C, allele of *SSIV-2* from CG173R; 1, allele of *PUL* from Guangzhan 63S) combinations had better ECQs.

**Conclusions:**

*SSIV-2* alleles significantly affect rice quality, especially the parameters relevant to gelatinized and thermal characteristics of starch (GC, PR, GT, PKV, BDV and SBV) under the same major genes (*Waxy* and *SSII-3*) background. It indicates that *SSIV-2* functions elongation of starch chain. These findings suggest that the effects of *SSIV-2* and its interaction with *AGPlar* and *PUL* are vital for rice quality breeding with the same major genes.

## Background

Rice (*Oryza sativa* L.) is not only one of the three major grain crops around the world, but also feeds more than half of the world’s population as staple food. In the past several decades, great breakthroughs have been made in rice yield and it has been greatly enhanced by green revolution and heterosis application. Rice qualities have now become the primary objectives for rice breeding programs. However, rice qualities are complicated traits, which are controlled by multiple genes and influenced simultaneously by environment. Although many studies have performed to understand the genetic basis of rice qualities (Li et al. 2014; Bello et al. 2019; Ji et al. 2019), their genetic mechanism are still unknown.

It is generally considered that eating and cooking qualities (ECQs) are the most crucial rice qualities, which are mainly determined by apparent amylose content (AAC), gel consistency (GC), and gelatinization temperature (GT) (Yan et al. 2011). In addition, because of its advantages of accurate, rapid and easy to perform, the rapid viscosity analyzer (RVA) profiles have been employed popularly to assess the rice ECQs in recent years (Bao and Xia 1999). Given that starch makes up approximately 90% of the rice grain, genes involved in starch biosynthesis are naturally expected to affect ECQs. Rice starch is composed of two polysaccharides: amylose and amylopectin, whose biosynthesis is involved in 18 genes (starch synthesis-related genes, SSRGs) and consists of four classes of enzymes: ADP-glucose pyrophosphorylase (AGP), soluble starch synthase (SS), starch branching enzyme (SBE), and starch debranching enzyme (DBE) (Tian et al. 2009). In the past decades, many researches focused on SSRGs how to regulate ECQs (Chiara et al. 2014; Xiang et al. 2017; Yang et al. 2018) and the accumulated conclusions showed that *Waxy* (*Wx*) encoding the granule-bound starch synthase I (GBSSI) and *SSII-3*(*SSIIa* /*ALK*) encoding SSIIa mainly controlled AAC (Wang et al. 1995; Xiang et al. 2017) and GT (Umemoto and Aoki 2005), respectively. However, the existing studies also revealed that ECQs were still quite different although the varieties with the same *Wx* and *SSII-3* (Yan et al. 2010). Therefore, there still are many challenging questions waiting for figuring out, such as the effects of minor genes and their interactions on ECQs.

*ADPglucose pyrophorylase large unit (AGPlar)* is responsible for encoding a large subunit of ADPglucose pyrophorylase (AGP), which is the first rate-limiting enzyme in starch biosynthesis. Overexpressing *AGPlar* could increase significantly the yield of cereals through boosting grain weight (Tuncel and Okita 2013). Meanwhile, *AGPlar* was in charge of the variations of several RVA parameters by an association analysis (Yan et al. 2011).

Compared with the other SSs, SSIV contains a highly similar C-terminal region, which includes the catalytic and starch-binding domains, and a unique N-terminal region with several long coiled-coil motifs that have been implicated in protein-protein interactions (Cao et al. 1999). Furthermore, Lu et al., (Lu et al. 2018) revealed that the specific N-terminal region of SSIV not only targeted itself to the correct subcellular location but also was responsible for forming the correct granule morphology. Taking Arabidopsis (*Arabidopsis thaliana*) as main experimental material, previously numerous researches have investigated the functions of SSIV (Seung et al. 2016; Malinova et al. 2017; Lu et al. 2018), and illuminated that SSIV had a minor impact on either the structure of amylopectin or the synthesis of amylose (Isaac et al. 2007). In immature leaves, however, when SSIV was absent, there was no starch granule, and presumably, the other SSs could not make use of ADP-glucose resulting in accumulating a very high level of ADP-glucose (Matilda et al. 2012; Paula et al. 2013). In addition, SS IV could be selectively involved in priming of starch granule formation, altering the number of starch granules and affecting the size and shape of granule (Isaac et al. 2007; Matilda et al. 2012). SSIV includes two isoforms in rice, SSIV-1 and SSIV-2 (Hirose and Terao 2004). *SSIV-2* locates on chromosome 5 and consists of 16 exons and 15 introns, which plays an important role in determining starch granule morphology and maintaining amyloplast envelope structure (Singh et al. 2017). Spatio-temporal expression patterns analysis showed that *SSIV-2* was mainly expressed in leaves and expressed consistently during grain filling, the transcript level of *SSIV-2* in endosperm was higher than that in pericarp. Moreover, it contributed significantly to grain chalkiness (Hirose and Terao 2004).

*Pullulanase* (*PUL*) is one of the DBEs, expressed only in the endosperm and responsible for modifying the starch to a perfect structure at the last stage of its biosynthesis. Its sequence variations in *indica* and *japonica* resulted in obvious differentiation in function that affected rice ECQs (Yan et al. 2010). The effect of *PUL* on rice ECQs could be masked by *Wx* and *PUL* played an important role in determining RVA profile parameters when *Wx* lost function (Yan et al. 2011).

Despite excellent progresses in defining the functions of SSIV have been made, however, the studies related genetic effects of *SSIV-2* on rice qualities and its interaction with *AGPlar* and *PUL* are limited. Hence, two backcross inbred lines (BIL) *SSIV-2* × *AGPlar* BIL, *SSIV-2* × *PUL* BIL came from BC_1_F_12_ generation and a near isogenic line (NIL-*SSIV-2*) were employed to investigated the genetic effects of *SSIV-2* and the interaction of *SSIV-2* × *AGPlar*, *SSIV-2* × *PUL* on rice qualities, aiming to provide useful information for molecular breeding of rice quality.

## Results

### Analysis on Genetic Background of Materials and Grouping of BILs and NILs

**Fig. 1:**
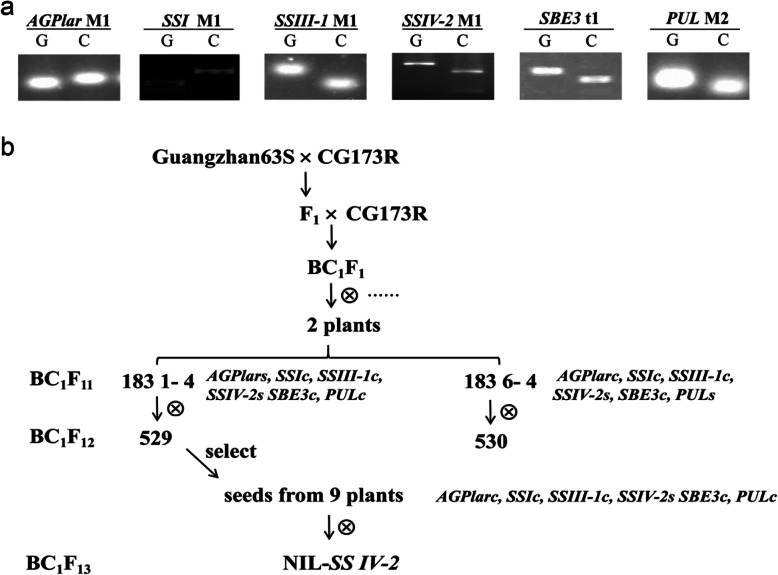
Genetic analysis of parents and flowchart of BILs and NIL population construction. **a**, Polymorphism gene loci of starch synthesis-related genes between two parents. G and C indicate homozygous for GZ63S and CG173R, respectively. **b**, Generation of BILs and NIL for starch synthesis-related genes. The italic letters followed by ‘c’ denote possessing the same alleles as CG173R, by “s” mean the alleles heterozygous

Polymorphism of eighteen SSRGs had been detected between two parents: Guangzhan (GZ) 63S and CG173R, and they differed in six genes (*AGPlar*, *SSI*, *SSIII-1*, *SSIV-2*, *SBE3* and *PUL*) (Fig. 1a). Then, two BILs were constructed under the background of CG173R to study the effects of *SSIV-2* and its interaction with *AGPlar* and *PUL* on rice ECQs. The first with 184 plants differed only in *AGPlar* and *SSIV-2* loci, another with 93 plants differed only in *PUL* and *SSIV-2* loci, which came from the BC_1_F_12_ generation. In order to further validate the effects of *SSIV-2*, seeds of 9 plants from BIL 529 that only had polymorphism at *SSIV-2* locus were identified and selected to develop the BC_1_F_13_, as a near isogenic line (NIL) at the *SSIV-2* locus with CG173R genetic background (NIL-*SS IV-2*) (Fig. 1b). Various genotypes were obtained according to different alleles among BIL and NIL populations, and were designated as follows: to the *AGPlar* locus, as type I (same as GZ63S), type II (same as CG173R) and type III (heterozygous), respectively; to the *SS IV-2* allele, as type G (came from GZ63S), type C (came from CG173R) and type H (heterozygous), respectively; to the *PUL* allele, as type 1 (same as GZ63S), type 2(same as CG173R) and type 3(heterozygous), respectively (Additional file 3:Fig. S1).

### Phenotypic Variation Analysis on Physicochemical Properties of BILs from BC_1_F_12_ and their Parents

**Fig. 2:**
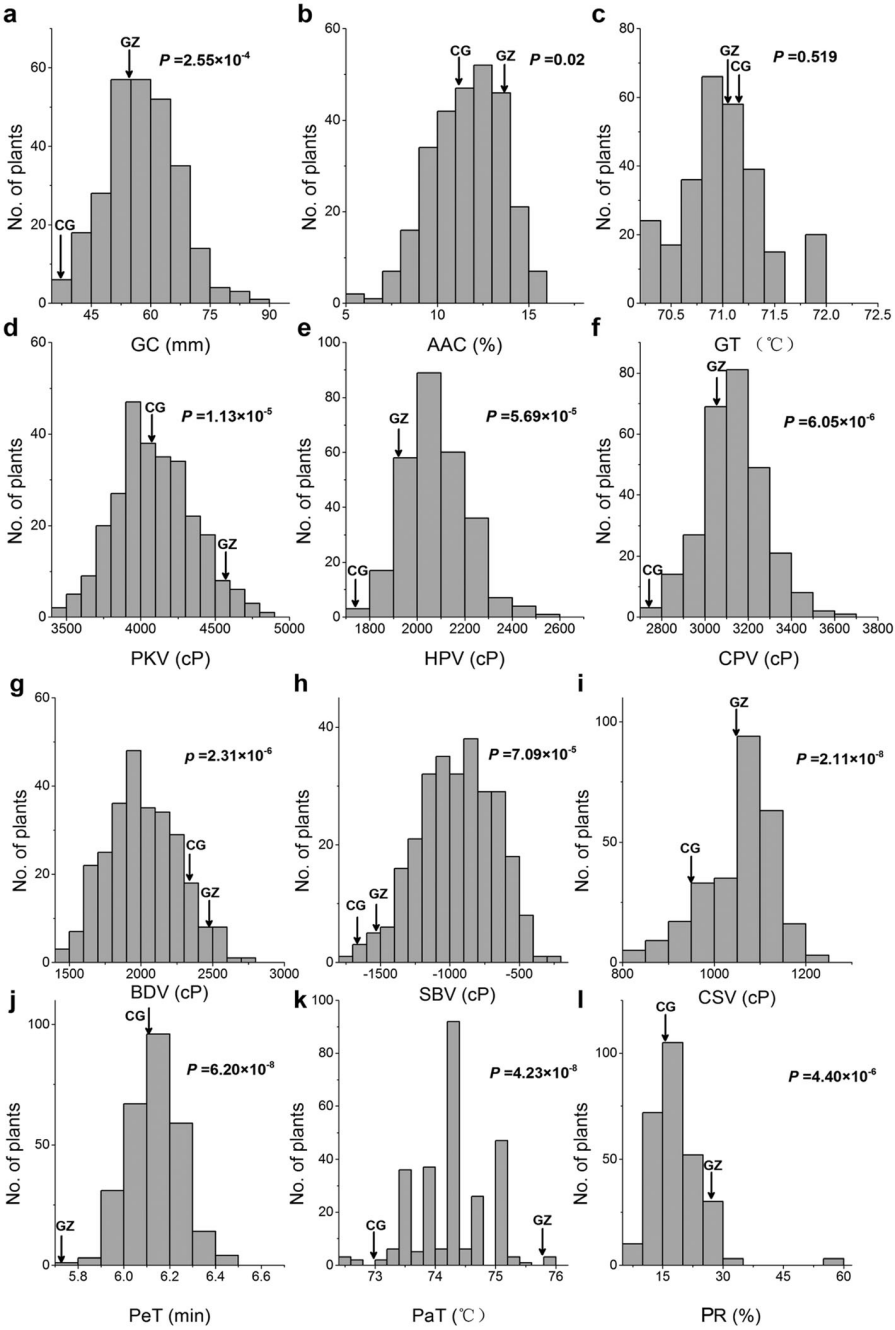
Distribution of the quality characters in BILs from BC_1_F_12_. GZ, GZ63S; CG, CG173R; **a** Gel consistency (GC); **b** Apparent amylose content (AAC); **c** Gelatinization temperature (GT); **d** Peak viscosity (PKV); **e** Hot paste viscosity (HPV); **f** Cool paste viscosity (CPV); **g** Breakdown viscosity (BDV); **h** Setback viscosity (SBV); **i** Consistence viscosity (CSV); **j** Peak time (PeT); **k** Pasting temperature (PaT); **l** Percent of retrogradation (PR). The *P* values, generated by student’s tests, denote the differences between GZ63S and CG173R

A *t* test was conducted to compare the difference of the two parents on physicochemical quality characters. The results clearly showed that the parents significantly differed on a majority of quality characters (Fig. 2), which were caused by their various SSRGs. The difference of GT between GZ63S and CG173R was not obvious because of same *SSII-3* allele for them. However, there was a significant difference on AAC (*P* = 0.02) although the parents had same *Wx* allele and belonged to low AAC (range 10–20%). This indicated that there were still other minor genes affecting AAC. Most of the ECQs and all the RVA parameters, such as GC, PKV, HPV and CPV, varied widely among the BC_1_F_12_. For example, the GC ranged from 36.00 mm to 87.00 mm, with an average of 57.03 mm, and with a variation coefficient of 15.82%. In contrast, the variations of GT and PeT were relatively narrow. GT were very close to 71 °C for most of the plants and the difference of PeT between the maximum and minimum is only 0.73 min. These results showed that the main ECQs characters were qualified for following genetic analysis.

### Correlation Analysis among Physicochemical Properties

In order to investigate the relationships among the tested physicochemical properties in BC_1_F_12_ population, a pairwise correlation analysis was carried out. The correlations between GC and all the RVA profile parameters, except BDV and SBV, were significantly positive (*p* < 0.01); And AAC was significantly correlated with HPV, CPV, SBV, PaT and PeT. Even though correlation analysis revealed that PR was significantly correlated with most of parameters, but all the correlation coefficients were small (r < 0.2) (Additional file 4:Fig. S2). On the other hand, significantly positive correlations were found between GT and PeT, PaT, and their correlation coefficients were 0.94 and 0.99, respectively.

### Effects of *SSIV-2* on Rice ECQs

**Fig. 3:**
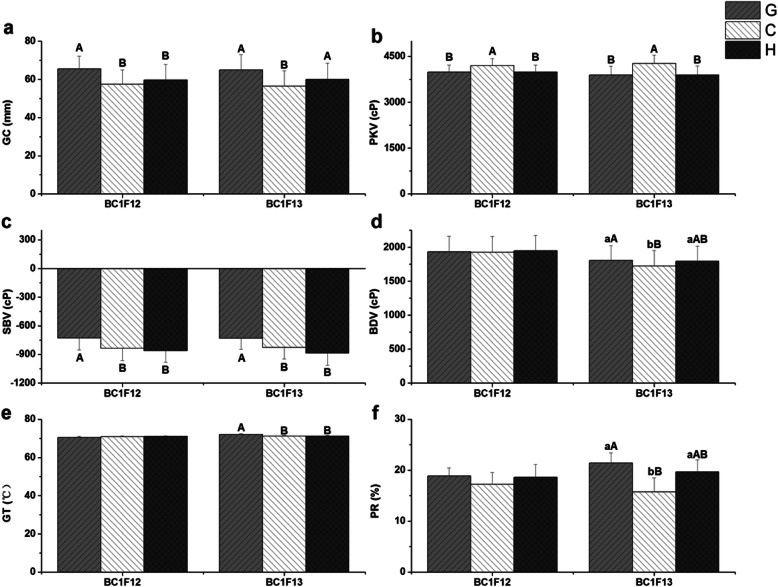
Comparison of starch physicochemical properties among different *SSIV-2* genotypes in BC_1_F_12_ and BC_1_F_13_ generation. **a** Gel consistency (GC); **b** Peak viscosity (PKV); **c** Setback viscosity (SBV); **d** Breakdown viscosity (BDV); **e** Gelatinization temperature (GT); **f** Percent of retrogradation (PR). Each column represents the mean ± standard deviation. Lowercase and capital letters above column denote significant at 0.05 and 0.01 levels, respectively. G denotes homozygous for GZ63S, C and H denotes homozygous for CG173R and heterozygote, respectively. Each sample was repeated twice

Among these investigated 184 plants from BC_1_F_12_ line 529, *SSIV-2* alleles were divided three genotypes: type G, type C and type H. Significant differences on GC, PKV and SBV among them were detected by analysis of variance (ANOVA). Multiple comparisons (Fig. 3) showed that GC of type G was the softest (65.50 mm), and obviously higher than that of type C and type H, whose GC was the hardest (57.41 mm) and intermediate (59.77 mm), respectively. And same phenomenon was observed in SBV. However, the PKV of type C was significantly higher than that of type G and type H (*p* < 0.01), but there was no notable difference between the PKV of type G and type H.

Afterwards, NIL-*SSIV-2*(BC_1_F_13_) with 341 plants was constructed in order to further confirm the effects of *SSIV-2* on rice ECQs. Consistently, GC, PKV and SBV of type G and type C significantly differed from each other. In addition, significant differences were also observed among different genotypes on BDV, GT and PR. Moreover, the samples with type G had significant lower PKV, in contrast, GC, SBV, BDV, GT and PR of type G significantly increased by 15.05%, 11.55%, 4.81%, 1.12% and 36.04%, respectively, compared with that of type C (*p* < 0.05 or *p* < 0.01) (Fig. 3). In order to make the results more powerful, comparisons between the plants of NIL-*SSIV-2* with GZ63S genotype (NIL-*SSIV-2*^*-GZ*^) and CG173R (recurrent parent) on these parameters were conducted. GC, PKV, CPV, SBV and PR of NIL-*SSIV-2*^*-GZ*^ altered significantly, nevertheless obvious changes of the other traits didn’t occurred in NIL-*SSIV-2*^*-GZ*^ (Table 1), which was basically consistent with the effects of *SSIV-2* in BC_1_F_12_ population*.* Taken together, the *SSIV-2* allele derived from GZ63S could significantly improve GC, SBV, BDV, GT and PR, but an opposite effect on PKV of starch. Therefore, *SSIV-2* allele derived from CG173R (type C) had better quality traits with lower GT, SBV and PR, but inferior traits with lower GC and BDV. Thus *SSIV-2* polymorphism has a great influence on rice ECQs under the same major genes (*Wx* and *SSII-3*) background.

**Table 1:** Comparison of physicochemical indexes between NIL-*SSIV-2* and recurrent parent

Materials	GC (mm)	AAC (%)	GT (°C)	PKV (cP)
NIL-*SS IV-2*^*-GZ*^	62.39 ± 5.61^**^	11.75 ± 1.33	71.64 ± 0.46	3768.82 ± 216.96^**^
CG173R	42.05 ± 1.15	11.13 ± 0.53	71.69 ± 0.58	4091.50 ± 29.50
	HPV (cP)	CPV (cP)	BDV (cP)	SBV (cP)
NIL-*SS IV-2*^*-GZ*^	2080.55 ± 115.42	3169.41 ± 141.08^**^	1852.64 ± 191.28	− 703.77 ± 183.91^**^
CG173R	2015.54 ± 19.45	2658.52 ± 20.52	2076.51 ± 10.54	− 1433.44 ± 29.31
	CSV (cP)	PeT (min)	PaT (°C)	PR (%)
NIL-*SS IV-2*^*-GZ*^	1088.86 ± 43.43	6.14 ± 0.11	75.48 ± 0.60	21.44 ± 6.98^**^
CG173R	943.55 ± 21.52	6.12 ± 0.03	74.62 ± 0.12	16.65 ± 0.15

*NIL-SS IV-2*^*-GZ*^ the plants of NIL-*SSIV-2* with GZ63S genotype, *GC* gel consistency, *AAC* apparent amylose content, *GT* Gelatinization temperature, *PKV* peak viscosity, *HPV* hot paste viscosity, *CPV* cool paste viscosity, *BDV* breakdown value, *SBV* consistence value, *SCV* setback value, *PeT* peak time, *PaT* pasting temperature, *PR* percent of retrogradation. ** indicates *P* < 0.01, according to Student’s *t*-test

### Combined Effects of *SSIV-2* and *AGPlar* Alleles on Rice ECQs

Due to *SSIV-2* and *AGPlar* alleles separated simultaneously in the tested line 529, their interactions on rice ECQs were able to further analyze by a split block design. The results demonstrated that interacting effects of *SSIV-2* and *AGPlar* alleles for ECQs were significant (*p* < 0.01) (Additional file 1: Table S1). The combined effects of *SSIV-2* and *AGPlar* alleles on rice ECQs should be further studied.

To the *AGPlar* locus, three genotypes, type I, type II and type III, were identified among the 184 plants, which resulted in nine combinations together with *SSIV-2* among the population of line 529 (Table 2). Multiple comparisons revealed that GC, AAC, GT and PR of the nine combinations significantly differed from each other (*p* < 0.05 or *p* < 0.01). The combination I-G and I-C possessed the softest gel (63.49 mm) and hardest gel (53.08 mm), respectively. Moreover, the AAC of I-G was similar with that of II-G, which was significantly higher than that of I-C and II-C. However, the I-C and II-G had similar GT, was significantly higher than that of I-G and II-C, with no significant difference from each other as well. PR of II-C (16.63%) was lower than that of I-G (19.20%), I-C (20.72%) and II-G (19.99%) (*p* < 0.05), whereas no significant differences among the next three combinations.

**Table 2:** Comparison of physicochemical indexes among different combinations of *AGPlar* and *SSIV-2* alleles

Genotype	Number of materials	GC (mm)	AAC (%)	GT (°C)	PR(%)
		Mean ± SD	Mean ± SD	Mean ± SD	Mean ± SD
I-G	25	63.49 ± 10.93aA	13.02 ± 1.51aAB	71.01 ± 0.53bcCDE	19.2 ± 3.75abcABCD
I-C	5	53.08 ± 2.48dF	12.03 ± 1.53cC	71.43 ± 0.33aA	20.72 ± 1.86aAB
I-H	9	56.63 ± 5.81cDE	12.86 ± 1.18abAB	70.88 ± 0.21cE	17.7 ± 4.79bcdBCD
II-G	13	61.74 ± 8.61abAB	13.18 ± 1.34aA	71.30 ± 0.46aAB	19.99 ± 4.56abABC
II-C	83	60.13 ± 8.27bBC	12.51 ± 1.45bBC	70.92 ± 0.36cDE	16.63 ± 7.25dCD
II-H	12	61.65 ± 6.19abAB	11.41 ± 1.28dD	71.11 ± 0.39bBCD	16.28 ± 6.02dD
III-G	9	57.33 ± 9.41cCDE	11.45 ± 1.34dD	71.02 ± 0.41bcCDE	17.21 ± 5.61cdCD
III-C	6	59.78 ± 7.59bBCD	12.77 ± 0.76abAB	71.12 ± 0.17bBCDE	21.61 ± 9.02aA
III-H	22	55.97 ± 8.02cEF	12.48 ± 1.19bBC	71.13 ± 0.31bBC	19.67 ± 5.22abcABC

Type I, II and III indicate homozygous for GZ63S, CG173R and heterozygote in *AGPlar* locus, respectively; Type G, C and H represent homozygous for GZ63S, CG173R and heterozygote in *SSIV-2* locus, respectively; *GC* gel consistency, *AAC* apparent amylose content, *GT* Gelatinization temperature, *PR* percent of retrogradation. Lowercase and capital letters denote significant at 0.05 and 0.01 levels, respectively

**Fig. 4:**
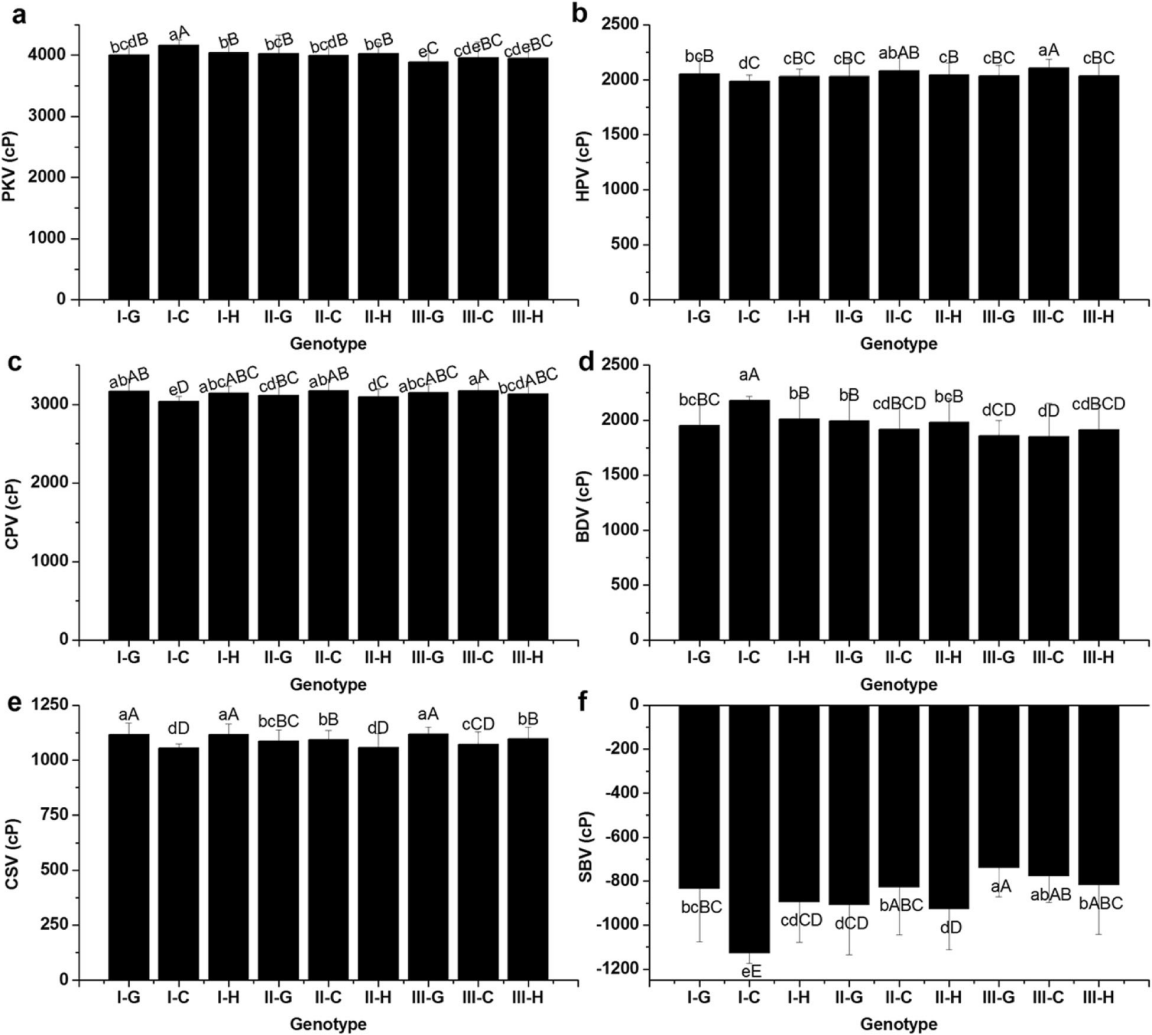
Comparison of RVA profile characteristics for different combinations of *SSIV-2* × *AGPlar*. **a** Peak viscosity (PKV); **b** Hot paste viscosity (HPV); **c** Cool paste viscosity (CPV); **d** Breakdown viscosity (BDV); **e** Consistence viscosity (CSV); **f** Setback viscosity (SBV); Type I, II and III indicate homozygous for GZ63S, CG173R and heterozygote in *AGPlar* locus, respectively; Type G, C and H represent homozygous for GZ63S, CG173R and heterozygote in *SSIV-2* locus, respectively; Each column represents the mean ± standard deviation. Lowercase and capital letters above column denote significant at 0.05 and 0.01 levels. Each sample was repeated twice

Significant differences on RVA profile characteristics were observed among the different combinations (Fig. 4). Specifically, I-C was significant higher in PKV (4166.65 cP) and BDV (2181.00 cP), but lower in HPV (1985.65 cP), CPV (3040.62 cP), CSV (1054.98 cP) and SBV (− 1126.02 cP) than the other three combinations: I-G, II-C and II-G (*p* < 0.05 or *p* < 0.01). Interestingly, it was observed that there were significant differences in GC, PKV and SCV between different *SSIV-2* genotypes under the background of type I, but not under the type II. Therefore, it implied that *AGPlar* had an epistatic effect on *SSIV-2*. Different combinations of *SSIV-2* and *AGPlar* genotypes played an important role in determining GC, AAC, GT, PR and all RVA profile characteristics except for PaT and PeT under the same major genes (*Wx* and *SSII-3*) background.

### Combined Effects of *SSIV-2* and *PUL* Alleles on Rice ECQs

To the BIL 530, 93 plants were divided into three groups by *PUL* alleles: type 1, type 2 and type 3; but only five combinations of *SSIV-2* and *PUL* alleles were obtained (Table 3). Results of multiple comparisons revealed that five combinations significantly differed from each other in GC and AAC. C-1 and C-3 (52.28 and 55.38 mm, respectively) possessed a medium GC (range 41–60 mm), and the others had a soft GC (> 61 mm). The AAC of G-2 (range 10.68–15.86%) was the highest, which was significantly higher than that of C-1 (range 5.20–13.06%).

**Table 3:** Comparison of GC and AAC among different combinations of *PUL* and *SSIV-2* alleles

Genotype	Number of materials	GC (mm)		AAC (%)	
		Mean ± SD	Range	Mean ± SD	Range
G-2	13	61.96 ± 8.6aA	47.50–77.00	13.18 ± 1.41aA	10.68–15.86
C-2	29	60.13 ± 8.27abA	43.00–87.00	12.51 ± 1.45aAB	8.71–15.62
H-2	12	61.54 ± 6.48aA	52.50–69.50	11.38 ± 1.35bB	8.83–13.71
C-1	24	52.28 ± 9.17cB	36.50–68.50	10.07 ± 1.52cC	5.20–13.06
C-3	15	55.38 ± 5.72bcAB	47.00–67.00	9.13 ± 1.95dC	5.54–12.70

Type G, C and H represent homozygous for GZ63S, CG173R and heterozygote in *SSIV-2* locus, respectively; Type 1, 2 and 3 indicate homozygous for GZ63S, CG173R and heterozygote in *PUL* locus, respectively; *GC* gel consistency, *AAC* apparent amylose content. Lowercase and capital letters denote significant at 0.05 and 0.01 levels, respectively

**Fig. 5:**
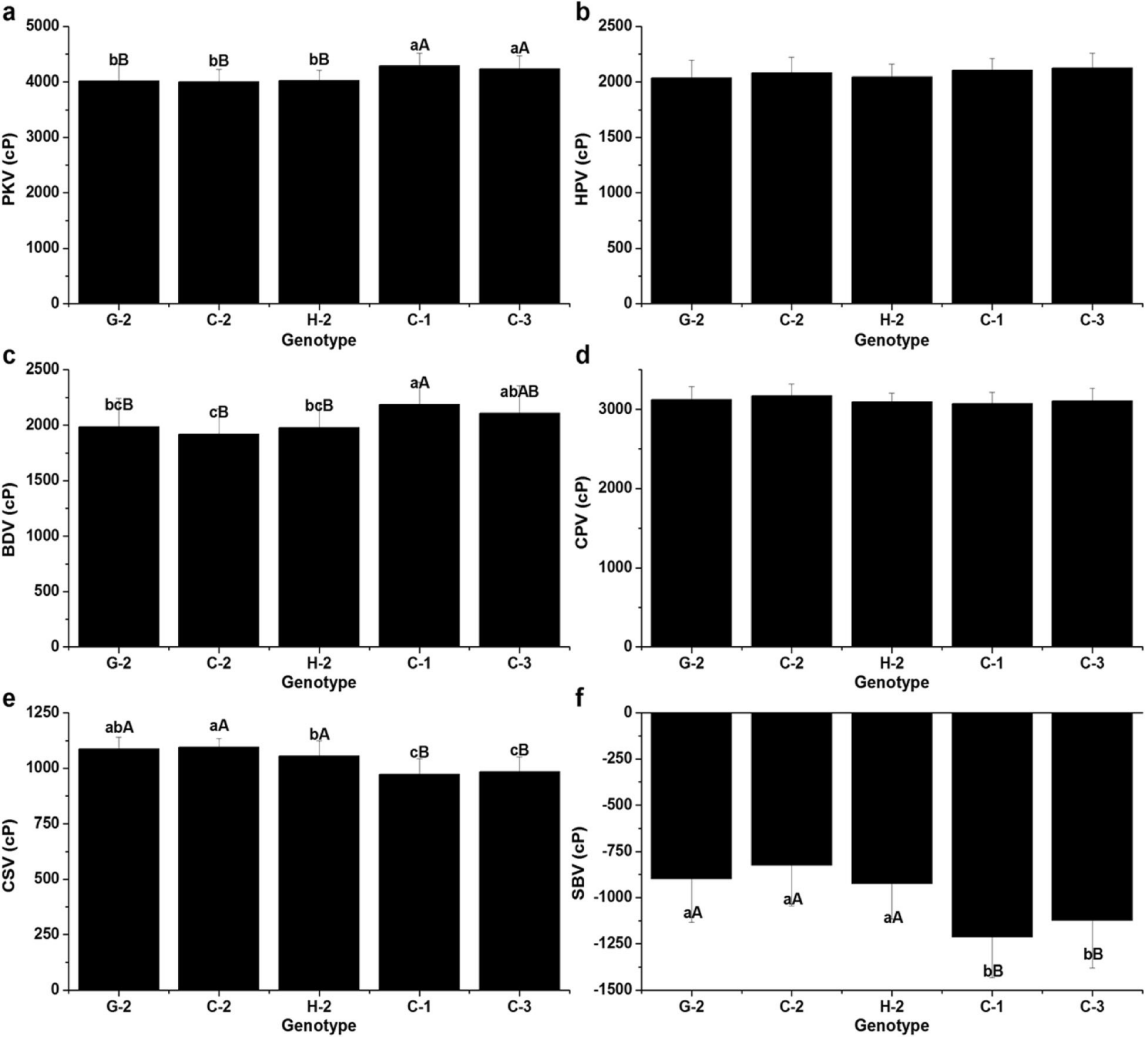
Comparison of RVA profile characteristics for different combinations of *SSIV-2* × *PUL*. **a** Peak viscosity (PKV); **b** Hot paste viscosity (HPV); **c** Breakdown viscosity (BDV); **d** Cool paste viscosity (CPV); **e** Consistence viscosity (CSV); **f** Setback viscosity (SBV); Each column represents the mean ± standard deviation. Type G, C and H represent homozygous for GZ63S, CG173R and heterozygote in *SSIV-2* locus, respectively; Type 1, 2 and 3 indicate homozygous for GZ63S, CG173R and heterozygote in *PUL* locus, respectively; Lowercase and capital letters above column denote significant at 0.05 and 0.01 levels

The combined effects of *SSIV-2* and *PUL* alleles were further investigated on rice RVA profile characteristics (Fig. 5). Significant difference was not detected on RVA profile characteristics between different *SSIV-2* genotypes under the same background of *PUL* (type 2). However, the PKV (4287.61 cP) and BDV (2185.01 cP) were greatly increased in C-1, while the CSV (972.13 cP) and SBV (− 1212.88 cP) were significantly decreased compared to that of G-2 and C-2. The results together indicated that combinations of *SSIV-2* and *PUL* alleles had remarkable impact on GC, AAC, PKV, BDV, CSV and SBV under the same major genes (*Wx* and *SSII-3*) background.

## Discussion

ECQs have now been considered as one of the most crucial targets in rice breeding. Because ECQs are typical quality-quantity traits, they are not only regulated by multiple genes but affected by environment simultaneously, which jointly makes it difficult to dissect the genetic mechanism of rice quality. The 18 genes involved in the synthesis of starch constitute a complex metabolic regulation network system. In addition to the major genes, the impacts of minor genes and their interactions on ECQs should be attached importance as well. Deciphering this sophisticated network will contribute to further improve rice qualities.

As one of bio-synthesis enzymes in this complex pathway, *SSIV-2* is responsible for encoding an isoform of SSIV. It was reported that SSIV could alter the number of starch granules and affect the morphology of granule in *Arabidopsis* (Isaac et al. 2007; Matilda et al. 2012). Singh et al. (2017) employed 150 diverse varieties to survey the origin and evolution of *SSIV-2* in rice and the results suggested that 9 SNPs were detected in *SSIV-2*. Its evolution in rice cultivars had a tri-phyletic origin with ancient haplotypes existing in *Aus* and *Indica* cultivars. Hirose and Terao (2004) analyzed the expression pattern of *SSIV-2* and found that it was expressed steadily during grain filling throughout the plant. In this work, a BIL with 184 plants was employed to explore the effects of *SSIV-2* on rice ECQs and the results illustrated that *SSIV-2* affected the GC, PKV and SBV, which was validated in NIL-*SSIV-2* population in the following year. In addition, we also observed that *SSIV-2* significantly affected the BDV, GT and PR. It should be attached importance to its effect on GT, small (only altered 1.12%) but significant (*p* < 0.01) (Fig. 3e), which was consistent with the report of Tian et al. (2009), that *SSIV-2* was minor gene affecting GT. Yang (2018) employed 63 glutinous rice to identify the genetic effects of 17 SSRGs on rice ECQs by association analysis and the results indicated that *SSIV-2* was involved in regulating GC, and with the same conclusion in our study as well (Fig. 3a). The conclusion should be accurate because relatively large populations (a BIL with 184 plants and a NIL with 341 plants) were used to study the effects of *SSIV-2*. Therefore, we can conclude that *SSIV-2* plays an important role in determining GC, GT, PKV, BDV and SBV (Fig. 3, Table 1) under the same major genes (*Wx* and *SSII-3*) background. *SSIV-2* significantly affects the parameters relevant to gelatinized and thermal characteristics of starch and it indicates that *SSIV-2* functions elongation of starch chain. Type C of *SSIV-2* has better quality traits with lower GT, SBV and PR. Thus, we should focus on selecting favorable allele on the *SSIV-2* locus (type C) in order to improve rice ECQs.

It is accepted that AGP is responsible for converting the G-1-P to ADGP, A precusor substance of the synthesis of starch. The *AGPlar* encodes a large submit of AGP, and naturally affects rice ECQs. As reported in previous articles, the *AGPlar* was found to have an impact on BDV, SBV and PeT in diverse glutinous varieties (Yan et al. 2011), and was also one of minor genes affecting AAC (Tian et al. 2009). The analysis on interactions of *SSIV-2* and *AGPlar* on ECQs demonstrated that different *SSIV-2* alleles had significant phenotypic differences on GC, PKV and CSV under the background of *AGPlar-*type I. Similar phenomena, however, were not observed under *AGPlar-*type II, which suggests that the effects of *SSIV-2* on ECQs might be masked by *AGPlar-*type II. Thus, *AGPlar-*type II has an epistatic effect on *SSIV-2*. GC of combination I-G (63.49 mm) and I-C (53.08 mm) altered significantly, belonged to soft (≥60 mm) and intermediate (41–60 mm), respectively. In addition, the combined effects of *SSIV-2* × *AGPlar* had an impact on AAC, GT, PR and all RVA profile parameters (Fig. 4, Table 2) except for PeT and PaT. I-C possessed the highest BDV (> 1000 cP) and the lowest SBV (< 250 cP) simultaneously, compared with other combinations. Thus it had better ECQs according to the previous paper (Wu et al. 2001). The *PUL* is one of vital important genes among the amylopectin synthesis pathway. Considerable researches on *PUL* were carried out and showed that PUL was involved in the biosynthesis of amylopectin in rice, and played an essential role in helping ISA to form amylopectin multiple-cluster structure (Kubo et al. 1999). Fujita et al. (2009) revealed that the short chain (DP < 13) would increase when PUL lost function and the average chain length of B2–3 chains increased by 3 glucose residues in *PUL* mutants compared with wild-type. Previous works showed that *PUL* was a minor gene affecting AAC (Tian et al. 2009), but as a major gene controlling RVA profile parameters in glutinous rice (Yan et al. 2011). However, in our study, no characters were detected to be correlated with *PUL* (data not shown); this result may be caused by the experimental materials (non-glutinous rice) and the effects of *PUL* might be masked by *Wx* (Yan et al. 2011). To date, numerous researches revealed that *PUL* interacted with many SSRGs on affecting rice ECQs (Yan et al. 2011; Zhang 2011; Yang 2018), for example, *PUL* interacted with *SSIV-2* on GC, with *SSII-3* on HPV, CPV, CSV (Yang 2018) and GT (Ying et al., 2006), with *SSI*, *SBE1* and *ISA* on BDV (Yan et al. 2011). In present study, five combinations were identified in *SSIV-2* × *PUL* BIL population and their combined effects on ECQs were investigated. The results illustrated that the GC altered significantly among different combinations (Table 3), which is consistent with the previous results of association analysis reported by Yang (2018). Apart from GC, the combined effects of *SSIV-2* and *PUL* greatly affected the AAC, PKV, CPV, CSV and SBV (Fig. 5), and C-1 with lower AAC, the highest BDV (> 1000 cP) and the lowest SBV (< 250 cP). It suggests that the interaction of *SSIV-2* and *PUL* play an important role in determining ECQs as well and C-1 has relatively better ECQs among the detected five combinations according to the report of Xiang et al. (2017).

Taken together, *SSIV-2* has a significant influence on GC, PR, PKV, BDV and SBV. *SSIV-2* allele derived from CG173R (type C) has better quality traits with lower GT, SBV and PR. Moreover, *AGPlar-*type II has an epistatic effect on *SSIV-2*. I-C possesses high BDV (> 1000 cP) and low SBV (< 250 cP). Combined effects of *SSIV-2* and *PUL* significantly affect the AAC, PKV, CPV, CSV and SBV; and C-1 with low AAC, high BDV (> 1000 cP) and low SBV (< 250 cP). The effects of different combinations of *SSIV-2* × *AGPlar*, *SSIV-2* × *PUL* on ECQs are significant different, which should be taken into consideration in rice quality breeding. Unfortunately, we didn’t get the combinations of *SSIV-2* and *PUL* alleles under the same background of *PUL* (type 1), in other words, without the combinations under the same genetic background of *PUL* with GZ63S. The conclusions will be in favor of us to understand the complicated genetic network of rice quality, especially the effects of minor gene *SSIV-2*.

## Materials and Methods

### Materials

GZ63S (*Oryza sativa ssp. indica*, a photo-thermo-sensitive genic male sterile line) and CG173R (potential restorer line), were used as the donor and recipient parents respectively to develop the backcross inbred lines (BILs) population. Line 529 with 184 plants and line 530 with 93 plants which came from the BC_1_F_12_ generation were harvested as experimental materials in 2017. Selecting seeds from BC_1_F_12_ to develop a set of near isogenic lines (NILs) with different *SSIV-2* alleles (NIL-*SSIV-2*) under same genetic background, and a total of 341 plants were collected from NIL-*SSIV-2* as experimental materials in 2018. All plants were grown in an experimental field at the Southwest University of Science and Technology under natural environmental conditions with same conventional cultivating method. Mature rice grains were harvested, air dried, and stored at room temperature for 3 months before their ECQs measurements.

## Methods

### DNA Extraction

About 100 mg rice young leaves was collected from each plant and ground using a Fastprep Sample Rapid Crushing System (MP Biomedicals, Santa Ana, CA, USA). DNA was extracted by using the CTAB method (Murray and Thompson 1980).

### Gene Genotyping

A total of 15 μL reaction mixtures using the Golden Easy PCR System (TIANGEN, Beijing, China), according to manufacturer’s instructions, were used to identify the genotypes of 18 SSRGs alleles by employing the primers designed according to Tian et al. (2009) and Cai et al. (2002). Detailed primer information was showed in Additional file 2: Table S2. All of the primers were synthesized by Sangon Biotech (Shanghai, China). Polymerase chain reactions (PCR) were carried out by using an Eppendorf Thermal Cycler (Mastercycler® nexus GSX1, Germany). 5 μL of each PCR product of CAPS marker was digested with corresponding restriction endonuclease in a total volume of 15 μL reaction mixture including 1.5 μL of 10 × buffer, 5 units of restriction endonuclease and sterile molecular biology grade water. Then the reaction was performed at 37 °C for 3–3.5 h. All the amplified products were detected on a 3% agarose gel in 0.5 × Tris-Borate EDTA (TBE) buffer using GreenView (Applied BioProbes, Rockville, MD, USA).

### Measurement of Physical and Chemical Indexes of Starch

Polished rice were milled into powder according to the protocol of Xiang et al. (2017). AAC and GC was measured according to the standard of Chinese Ministry of Agriculture, NY/T2639–2014, and Chinese national standards, GB/T 17891–1999, respectively. GT was measured by using a differential scanning calorimetry (DSC) according to methods reported previously (Xu et al. 2019). In order to measure PR, the gelatinized starch was stored at 4 °C for 7 days, afterwards, the starch was equilibrated to room temperature for 1 h and then with the same thermal program as the measurement of GT. PR was calculated by the following formula:

$$ \mathrm{PR}=\varDelta \mathrm{Hr}\;\left(\mathrm{enthalpy}\ \mathrm{of}\ \mathrm{retrogradation}\right)\ast 100\%/\varDelta \mathrm{Hg}\;\left(\mathrm{enthalpy}\ \mathrm{of}\ \mathrm{gelatinization}\right) $$

Each index of per sample was measured in duplicate (parallel test).

### Determination of RVA Profile Characteristics

A RVA (Model No. RVA4500, NewPortSci. Co. Warriewood, Australia) was used to assess RVA profile characteristics in accordance with the manufacturer’s instructions and performed according to the American Association of Cereal Chemists standard method AACC61–02 with duplicate for each sample. Three original parameters obtained from the RVA profile as following: peak viscosity (PKV), hot paste viscosity (HPV), cool paste viscosity (CPV); and three secondary parameters: breakdown viscosity (BDV=PKV-HPV), setback viscosity (SBV=CPV-PKV), consistence viscosity (CSV=CPV-HPV), were used to evaluate rice starch viscosity characteristics. In addition, rice starch viscosity characteristics also included pasting temperature (PaT) and pasting time (PeT). Each starch sample was repeated twice.

### Data Statistics and Analysis

Pearson correlation analysis among physical and chemical quality characters and ANOVA were performed by using the Statistical Product and Service Solutions software version 19 (SPSS, https://www.ibm.com/analytics/cn/zh/technology/spss/) after data being classified into groups according to the genotyping results. Multiple comparisons were performed by employing the Duncan’s method in SPSS 19.

## Supplementary information

**Additional file 1: Table S1.** The results of split block design for *SSIV-2* and *AGPlar* alleles.

**Additional file 2: Table S2.** Primer sequences of molecular markers used to identify the genotypes of 18 SSRGs in rice.

**Additional file 3: Figure S1.** Segregation of three target genes among partial tested materials. Type I, G, 1; II, C, 2; and III, H, 3 indicate homozygous for GZ63S, CG173R and heterozygote in *AGPlar, SSIV-2, PUL* loci, respectively.

**Additional file 4: Figure S2.** Phenotypic correlations among physical and chemical quality characters in RILs. Correlations with *P* <0.05 are in bold, while values with *P* <0.01 are in bold and underlined.

## Data Availability

Not applicable.
