# Supplementary material for: Genetic Effects of Soluble Starch Synthase IV-2 and It with ADPglucose Pyrophorylase Large Unit and Pullulanase on Rice Qualities
Source: Rice (N Y). 2020 Jul 13;13:46. doi: 10.1186/s12284-020-00409-0 (PMC7359214; doi:10.1186/s12284-020-00409-0)
Supplement: Supplementary file 1 — Additional file 1: Table S1. The results of split block design for SSIV-2 and AGPlar alleles. [file 12284_2020_409_MOESM1_ESM.docx]

**Table S1 The results of split block design for *SSIV-2* and *AGPlar* alleles.**

| Index of ECQs | *SSIV-2* | |  | *AGPlar* | |  | *SSIV-2*×*AGPlar* | |
| --- | --- | --- | --- | --- | --- | --- | --- | --- |
|  | F value | P value |  | F value | P value |  | F value | P value |
| AAC | 3.2650 | 0.0390 |  | 6.3990 | 0.0021 |  | 35.653 | 0.0001 |
| GC | 13.445 | 0.0001 |  | 14.516 | 0.0001 |  | 17.564 | 0.0001 |
| PKV | 5.6120 | 0.0039 |  | 26.738 | 0.0001 |  | 4.5620 | 0.0013 |
| HPV | 2.6480 | 0.0718 |  | 6.2340 | 0.0025 |  | 10.336 | 0.0001 |
| CPV | 1.9250 | 0.1470 |  | 4.4820 | 0.0127 |  | 17.416 | 0.0001 |
| BDV | 3.1470 | 0.0439 |  | 48.575 | 0.0001 |  | 12.633 | 0.0001 |
| CSV | 30.665 | 0.0001 |  | 10.845 | 0.0001 |  | 24.644 | 0.0001 |
| SBV | 9.3400 | 0.0001 |  | 54.745 | 0.0001 |  | 20.594 | 0.0001 |
| PaT | 1.1850 | 0.3065 |  | 0.5060 | 0.6036 |  | 7.2310 | 0.0001 |
| PeT | 3.1120 | 0.0454 |  | 25.284 | 0.0001 |  | 15.281 | 0.0001 |
| GT | 5.2120 | 0.0060 |  | 1.8540 | 0.1625 |  | 19.054 | 0.0001 |
| PR | 3.2820 | 0.0390 |  | 4.7840 | 0.0106 |  | 6.2770 | 0.0001 |

AAC, apparent amylose content; GC, gel consistency; PKV, peak viscosity; HPV, hot paste viscosity; CPV, cool paste viscosity; BDV, breakdown value; SCV, setback value; SBV, consistence value; PaT, pasting temperature; PeT, peak time; GT, Gelatinization temperature; PR, percent of retrogradation.
