# Supplementary material for: Genetic Effects of Soluble Starch Synthase IV-2 and It with ADPglucose Pyrophorylase Large Unit and Pullulanase on Rice Qualities
Source: Rice (N Y). 2020 Jul 13;13:46. doi: 10.1186/s12284-020-00409-0 (PMC7359214; doi:10.1186/s12284-020-00409-0)
Supplement: Supplementary file 2 — Additional file 2: Table S2. Primer sequences of molecular markers used to identify the genotypes of 18 SSRGs in rice. [file 12284_2020_409_MOESM2_ESM.docx]

**Table S2** **Primer sequences of molecular markers used to identify the genotypes of 18 SSRGs in rice**

| Gene | Molecular marker | Sequence of primers (5′- to 3′) | Marker type | Reference |
| --- | --- | --- | --- | --- |
| *AGPlar* | *AGPlar* M1 | 1. CGTTCAGGTTCAGGCAATCA   [R]GGAAGGGTGGTGATGTGGAG | STS | Tian *et al*., 2009 |
|  | *AGPlar* M2 | [F] GCGTGAACTGAACATCCATCT  [R] GGTTCAAGCCTTCAGGTCAG | CAPS (*Tsp45* I ) | Tian *et al*., 2009 |
| *AGPiso* | *AGPiso* M2 | [F] CAATCGCTGCCATCGGTTG  [R] TTCCACATCGTTAGGTACACG | STS | Tian *et al*., 2009 |
|  | *AGPiso* M3 | [F] TGGAATGGGAACTCTATTATTGG  [R] TCCCAACCTCTACCTTCAAATG | CAPs(*Eco*R I) | Tian *et al*., 2009 |
| *AGPsma* | *AGPsma* M1 | [F] TCTATTCTCAGCCCTCCAACC  [R] GTGTGTTTAGAGGTGCTTTTCG | STS | Tian *et al*., 2009 |
|  | *AGPsma* M2 | [F] GTGTGTTTAGAGGTGCTTTTCG  [R] TATCTTCCCAGTAACCATCA | STS | Tian *et al*., 2009 |
| *GBSSII* | *GBSSII* | [F] TTGCTGCGAATTATCTGCG  [R] ACCTCCTCCCACTTCTTTGC | STS | Tian *et al*., 2009 |
| *SSI* | *SSI* M1 | [F] GGTAGGGTAGGTCAATCTGGC  [R] ATAGAGAAGACAATGTGGCAACC | CAPs(*Nru* I) | Tian *et al*., 2009 |
|  | *SSI* M2 | [F] CTTCTATCCATTCCTTAATCCCA  [R] ATGCTATTGATGTTAAGAGGGC | STS | Tian *et al*., 2009 |
|  | *SSI* M3 | [F] GACCCACCTCGCTATCTGTTG  [R]GGAAACACCAGACATCAACCAG | CAPs(*Apa* I ) | Tian *et al*., 2009 |
| *SSII-1* | *SSII-1* M1 | [F] CACCCCACCGTTCTACTATGC  [R] TCCATAGTTTCATTGAGATTGCTC | STS | Tian *et al*., 2009 |
|  | *SSII-1* M2 | [F]CAAGTTGGTGACGATAGTGATGA  [R]AACAGAGCCTCCATTACCTTTAC | CAPs(*Age* I) | Tian *et al*., 2009 |
|  | *SSII-1* M3 | [F] AGAGATCAAATCGTGGAAC  [R] TGGAGTGAAGTAGTGGAAT | STS | Tian *et al*., 2009 |
|  | *SSII-1* M4 | [F] ATCTTTAGACGATTAGCG  [R] AAGTCACAAGTAGAAGGG | STS | Tian *et al*., 2009 |
| *SSII-2* | *SSII-2* M1 | [F] AGATTTGAACTCAGGACTTGGTG  [R] TCTATGGGCTCTATCCTTACTAGG | STS | Tian *et al*., 2009 |
|  | *SSII-2* M2 | [F] CGCTCGTTGCCTAGCTAGC  [R] GGCGAGGAAGCGATTGCC | STS | Tian *et al*., 2009 |
|  | *SSII-2* M3 | [F] ACAGTATGTTTGCCTCAGCG  [R] GTAAATCCACCCAGCCAGTC | STS | Tian *et al*., 2009 |
| *SSII-3* | *SSII-3* M1 | [F] CCAATACCGTAAACTAGCGACTATG  [R] TACAGGTAGAATGGCAGTGGTG | STS | Tian *et al*., 2009 |
|  | *SSII-3* M2 | [F] GGTTCTCGGTGAAGATGGC  [R] GTGGTCCCAGCTGAGGTCC | CAPs(*Ban* II) | Tian *et al*., 2009 |
|  | *SSII-3* M3 | [F] AACTGACTCATACACGGATAACG  [R] CACGCACGAACGGAAACC | CAPs(*Nhe* I) | Tian *et al*., 2009 |
| *SSIII-1* | *SSIII-1* M1 | [F] AAGAAGGGAAGGGAGTCAGC  [R] GCCATCTCCATTGCCAGC | STS | Tian *et al*., 2009 |
|  | *SSIII-1* M2 | [F] CAAGCAATGATTCAGGCACA  [R] GGAGACAGGAGCAAAAGGC | CAPs(*Eco R*I) | Tian *et al*., 2009 |
|  | *SSIII-1* M3 | [F] CAAATCAACTGTAAGTGCTGGAG  [R] GAGAACGGAGAAAATGGCAT | CAPs*(Nde* I) | Tian *et al*., 2009 |
|  | *SSIII-1* t1 | [F] GGAGCAATAGGTGGTTCAA  [R] GCCAAATCTACTCTCGTCA | CAPs(*Eco R*72I) | Tian *et al*., 2009 |
| *SSIII-2* | *SSIII-2* M1 | [F] AAGTCCTTCGGCTTACTATTCC  [R] GGAGAAGGAACATAACAGGGAC | CAPs(*Xba* I) | Tian *et al*., 2009 |
|  | *SSIII-2* M2 | [F] AAGCAATGTAAGTTCAAGTAGC  [R] GATTAGGGATGATGGTTTTC | STS | Tian *et al*., 2009 |
| *SSIV-1* | *SSIV-1* M1 | [F] CATTGTGTCTTGAAGTCTGTGCT  [R] CGATGGGTTAGTGCTGTGG | CAPs(*Nde* I) | Tian *et al*., 2009 |
| *SSIV-2* | *SSIV-2* M1 | [F] CTTCTGATTGATGGTTGGTTGC  [R] GGAAGAATAATCTCTACTAGGTGGC | CAPs(*Sph*I) | Tian *et al*., 2009 |
|  | *SSIV-2* M2 | 1. TTCCCTTGGTGGTGCGTG   [R] TAAAGCGTTCCGACAGTA | STS | Tian *et al*., 2009 |
|  | *SSIV-2* M3 | [F] TCAAGTATGGTTTACCTATG  [R] TTTCCCAATGACTTCTAA | CAPs(*Eco72*I) | Tian *et al*., 2009 |
| *SBE1* | *SBE1* M1 | [F] GCTACATAACACGCATACAAAGT  [R] AGACAAAAGCGAAAGGTAATGAG | STS | Tian *et al*., 2009 |
|  | *SBE1* M2 | [F] GTGGGGAAAACAAGTAAGTCTG  [R] AGTTCCATCAGAAGAATCAGGG | STS | Tian *et al*., 2009 |
|  | *SBE1* M3 | [F] GGAAATGGGAGTCGCC  [R] CGAAGAAACCACGCTCA | STS | Tian *et al*., 2009 |
|  | *SBE1* M4 | [F] ATTGTTGCTGAAGATGTTT  [R] ACGGTTGATGGTAGGTG | CAPs(*Taq* I) | Tian *et al*., 2009 |
| *SBE3* | *SBE3* M1 | [F] AAGGTTAGCATTGGTTGGTGAG  [R] TCTCCTTGAACAGCGACAGC | STS | Tian *et al*., 2009 |
|  | *SBE3* t1 | [F] TTCCATTATTTCTTTGCA  [R] TATCCTCCCTGAACCAC | STS | Tian *et al*., 2009 |
| *SBE4* | *SBE4* M1 | [F] CACCAATTATATTAGCGTGCTCC  [R] CGTGGCTCTTGGCTCTCTTG | STS | Tian *et al*., 2009 |
|  | *SBE4* M2 | [F] CCATCACCTCAAATACATCACTC  [R] AGACTGGAATGCCCCTTAGG | STS | Tian *et al*., 2009 |
| *ISA* | *ISA* M1 | [F] ATAGATGCTAATGTGATGTGGC  [R] TGGTATAGGCACAACCGTAGA | STS | Tian *et al*., 2009 |
|  | *ISA* M2 | [F] ACAAGCACACGACACCTA  [R] CAACAAACCAAACTCATT | CAPs(*Hind* III) | Tian *et al*., 2009 |
|  | *ISA* M3 | [F] TGTGGGAATACCTTCAACTG  [R] ATAAAACCCTTACAGGCTTG | STS | Tian *et al*., 2009 |
| *PUL* | *PUL* M2 | [F] GACAACCGTCCGCTTTAGTTTC  [R] GCATTTGAGAGGGTTTGGATTC | STS | Tian *et al*., 2009 |
|  | *PUL* M6 | [F] ATTTAACTGTATGGACTGAG  [R] GATACCAACCAAACAAGA | STS | Tian *et al*., 2009 |
| *Wx* | *Wx* M1 | [F] CACAGCAACAGCTAGACAACCAC  [R] CACGACGACGGAGGGGAAC | STS | Tian *et al*., 2009 |
|  | PCR-ACCI | [F]GCTTCACTTCTCTGCTTGTG  [R] ATGATTTAACGAGAGTTAA | CAPs(*ACC*I) | Cai *et al*., 2002 |
|  | *Wx* EX10 | [F] GCATCACCGGCATCGTC  [R]GCTCCGGCCATGATGAGATG | CAPs(*Apa* I) | Tian *et al*., 2009 |
