# Supplementary material for: Genetic Effects of Soluble Starch Synthase IV-2 and It with ADPglucose Pyrophorylase Large Unit and Pullulanase on Rice Qualities
Source: Rice (N Y). 2020 Jul 13;13:46. doi: 10.1186/s12284-020-00409-0 (PMC7359214; doi:10.1186/s12284-020-00409-0)
Supplement: Supplementary file 3 — Additional file 3: Figure S1. Segregation of three target genes among partial tested materials. Type I, G, 1; II, C, 2; and III, H, 3 indicate homozygous for GZ63S, CG173R and heterozygote in AGPlar, SSIV-2, PUL loci, respectively. [file 12284_2020_409_MOESM3_ESM.docx]

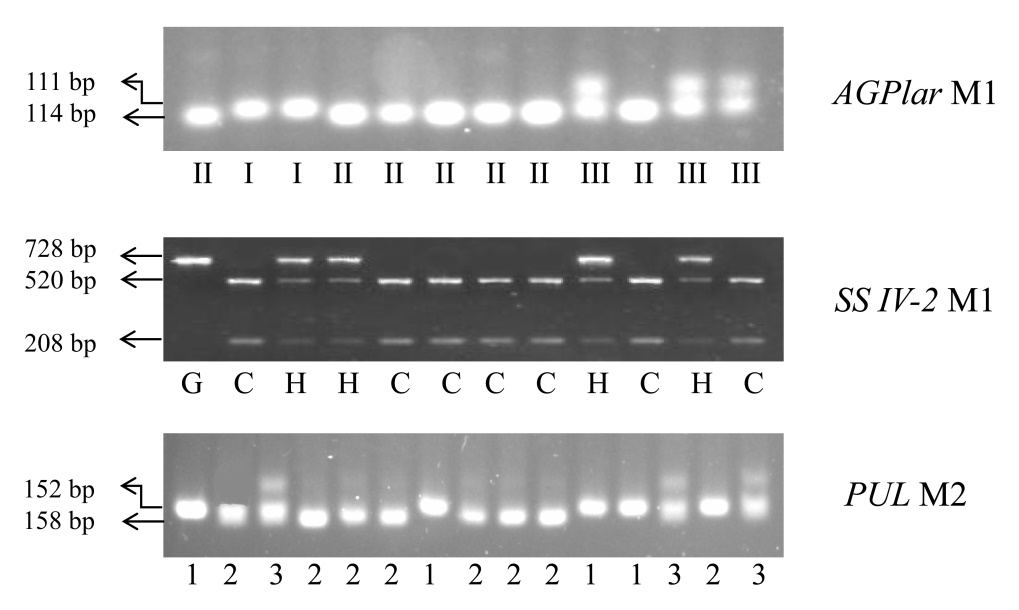


**Figure S1. Segregation of three target genes among partial tested materials.** Type I, G, 1; II, C, 2; and III, H, 3 indicate homozygous for GZ63S, CG173R and heterozygote in *AGPlar, SSIV-2, PUL* loci, respectively
