# Supplementary material for: Genetic Effects of Soluble Starch Synthase IV-2 and It with ADPglucose Pyrophorylase Large Unit and Pullulanase on Rice Qualities
Source: Rice (N Y). 2020 Jul 13;13:46. doi: 10.1186/s12284-020-00409-0 (PMC7359214; doi:10.1186/s12284-020-00409-0)
Supplement: Supplementary file 4 — Additional file 4: Figure S2. Phenotypic correlations among physical and chemical quality characters in RILs. Correlations with P <0.05 are in bold, while values with P <0.01 are in bold and underlined. [file 12284_2020_409_MOESM4_ESM.docx]

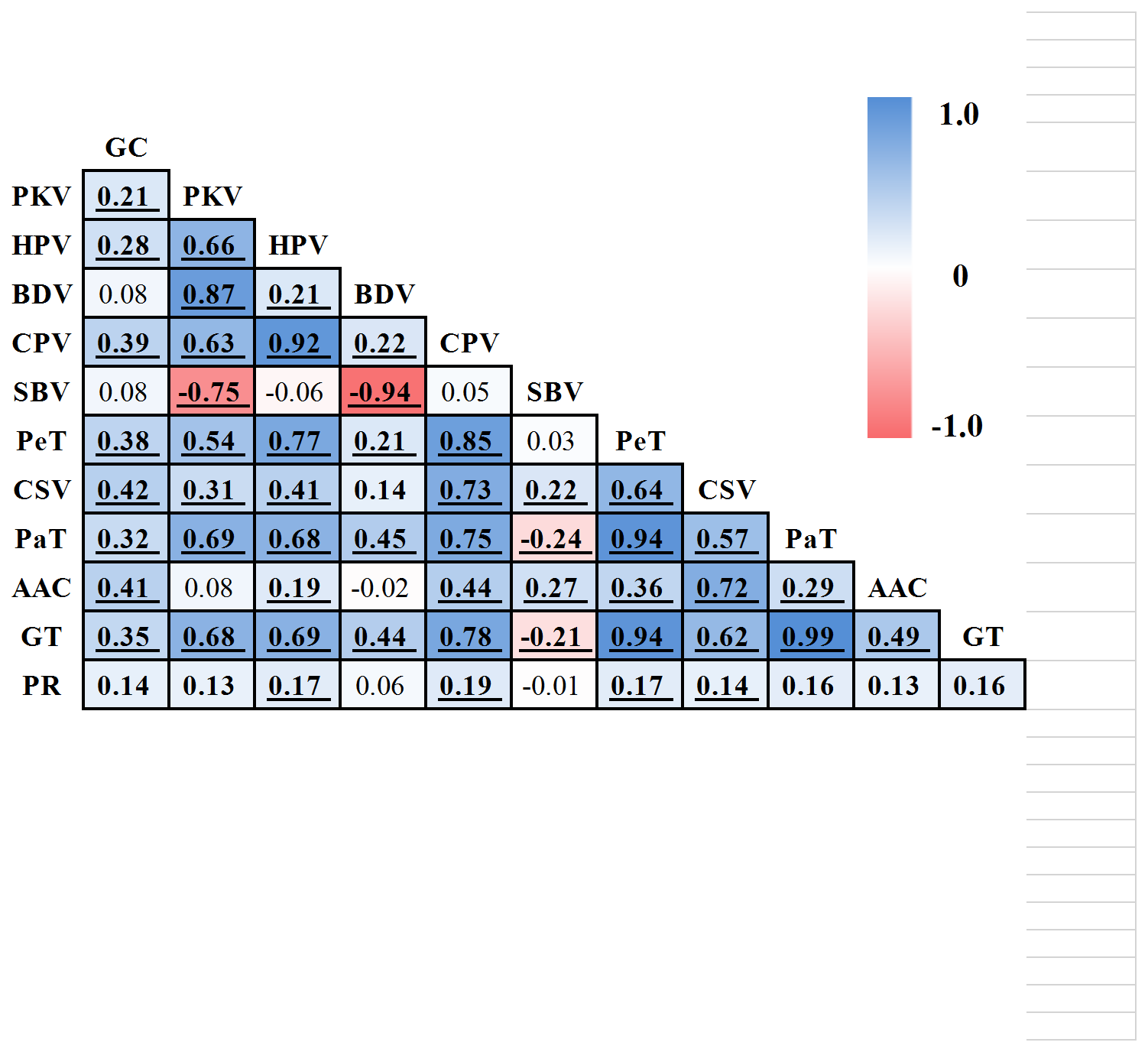


**Figure S2. Phenotypic correlations among physical and chemical quality characters in RILs.** Correlations with *P* <0.05 are in bold, while values with *P* <0.01 are in bold and underlined
